# Confusion and Anxiety Following Breast Density Notification: Fact or Fiction?

**DOI:** 10.3390/jcm9040955

**Published:** 2020-03-30

**Authors:** Evenda K. Dench, Ellie C. Darcey, Louise Keogh, Kirsty McLean, Sarah Pirikahu, Christobel Saunders, Sandra Thompson, Catherine Woulfe, Elizabeth Wylie, Jennifer Stone

**Affiliations:** 1 Centre for Genetic Origins of Health and Disease, School of Population and Global Health, University of Western Australia, 6009 Perth, Australia; evenda.dench@uwa.edu.au (E.K.D.); ellie.darcey@uwa.edu.au (E.C.D.); kirsty.mclean@uwa.edu.au (K.M.); sarah.pirikahu@uwa.edu.au (S.P.); woulfec@hotmail.com (C.W.); 2 Centre for Epidemiology and Biostatistics, The University of Melbourne, 3010 Melbourne, Victoria, Australia; l.keogh@unimelb.edu.au; 3 School of Medicine, The University of Western Australia, 6009 Perth, Western Australia, Australia; christobel.saunders@uwa.edu.au (C.S.); liz.wylie@health.wa.gov.au (E.W.); 4 Western Australian Centre for Rural Health, School of Population and Global Health, The University of Western Australia, 6009 Geraldton, Western Australia, Australia; sandra.thompson@uwa.edu.au; 5 BreastScreen Western Australia, Women and Newborn Health Service, 6000 Perth, Western Australia, Australia; 6 The Medical Research Foundation, Royal Perth Hospital, 6000 Perth, Western Australia, Australia

**Keywords:** breast density, breast cancer screening, anxiety, screening intentions, breast density notification

## Abstract

In the absence of evidence-based screening recommendations for women with dense breasts, it is important to know if breast density notification increases women’s anxiety. This study describes psychological reactions and future screening intentions of women attending a public mammographic screening program in Western Australia. Two-thirds of notified women indicated that knowing their breast density made them feel informed, 21% described feeling anxious, and 23% confused. Of the notified women who reported anxiety, 96% intended to re-screen when due (compared to 91% of all notified women and 93% of controls; *p* = 0.007 and *p* < 0.001, respectively). In summary, reported anxiety (following breast density notification) appears to increase women’s intentions for future screening, not the reverse.

## 1. Introduction

Routine breast density notification has improved women’s knowledge of the reduced sensitivity of mammography in dense breasts and, to a lesser extent, increased risk of breast cancer [1,2]. Growing awareness and knowledge of breast density is largely due to consumer advocacy and increasing breast density notification legislation in the United States (US) and Canada. Whilst there is considerable potential for routine measurement of breast density to improve breast cancer outcomes [3], there are currently no evidence-based additional screening recommendations for women with dense breasts. There is concern that breast density notification in the absence of further screening recommendations may inflate patients’ anxiety without improving health [4]. Anxiety appears to be one of the key arguments against breast density notification; however, there is limited research into women’s psychological, social and behavioural response to breast density notification.

In Australia, mammographic screening is free to all women aged 40 and over and the BreastScreen Western Australia (WA) program, which screens approximately 125,000 women each year, has been notifying women if they have dense breasts for over a decade. Sample text is provided in Supplementary Figure S1. Concurrent work assessed the knowledge and awareness of breast density within the publicly funded BreastScreen WA program [5] and explored the actions of women who were notified that they had dense breasts [6] via an online survey of around 6000 women. It was found that most women have heard of breast density [5] and that about half of women who are notified that they have dense breasts consult their doctor. Of those, around 20% go on to have supplemental ultrasounds due to their breast density [6]. These estimates are useful for other publicly funded screening programs to assess the additional costs associated with breast density notification; however, they do not factor the associated psychological benefits and harms of a woman knowing she has dense breasts. Nor do they factor the social and behavioural response to breast density notification, for example a woman’s future intention to re-screen.

In this study, we aim to quantify women’s self-reported anxiety and confusion around breast density notification and how these reactions impact women’s future screening intentions. We explore what it means to be anxious and/or confused about breast density via qualitative analysis of open-ended responses to questions about how knowing breast density makes women feel. We also thematically analyse responses to open-ended responses about women’s reasons for attending future mammographic screening.

## 2. Materials and Methods

### 2.1. Survey Development

The survey collected information on key themes identified by a consumer consultation. Validated questions addressing each of the themes were identified from a comprehensive literature review [2,7,8] and shortlisted by two consumer reference groups. This article presents an analysis of women’s responses to the 3 survey questions, plus 2 open-ended questions designed to elicit data on women’s psychological reaction and future screening intentions post receiving dense breast notification. The questions were: “Knowing my breast density makes me feel/would make me feel…<informed>, <confused>, <anxious>” followed by “Would you like to comment?”, “Would you say that your experience of being told your breast tissue is dense has put you off breast screening in the future?”, “Do you intend to have another mammogram with BreastScreen WA when you are next due?” and “Can you comment on the reason for your intention?” In addition, demographic information including year of birth, ethnicity and postcode was collected.

### 2.2. Recruitment

The survey was administered to all women who attended BreastScreen WA from 21 November, 2017, to 19 April, 2018, who received a routine results letter and provided an email address (~70% of women). Emails were sent to 30,566 eligible women, where 1150 emails bounced back and 72 women unsubscribed. The online version of the survey was administered using the software program Qualtrics and a link to the survey was provided within the email invitation. The link provided to notified women was different from that provided to non-notified women; therefore, breast density notification status was known for each participant. Otherwise, responses were anonymous unless women opted to provide their contact details for future contact. The survey took approximately 5–10 min to complete. Of the 29,416 who received email invitations, 6922 responded to the survey resulting in a 23.5% response rate (See Supplemental Figure S2).

### 2.3. Selection of Subjects

**Figure 1:**
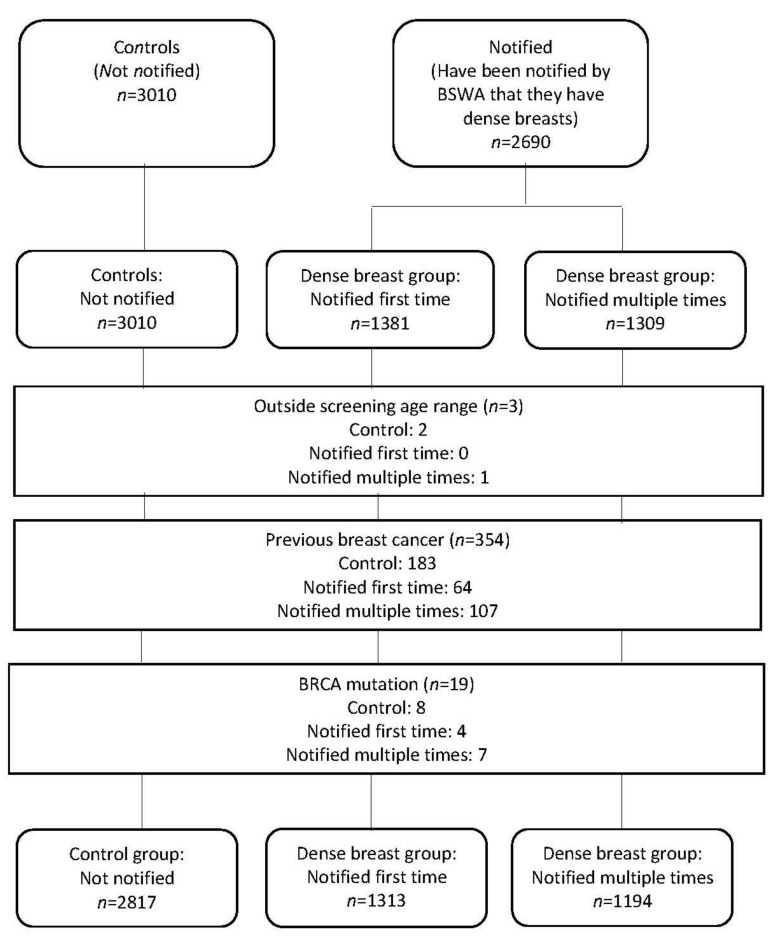
Flowchart of breast density survey respondents.

Respondents were categorised into groups based on their breast density notification status (according to their most recent results letter) and their response to “Have you ever been told by a health professional (e.g., BreastScreen WA) that your breast tissue is dense?” Controls were defined as women whose most recent results letter did not include breast density notification and responded either “No” or “I don’t know” (*n* = 3010). Women whose results letter included breast density notification and responded that they either had been told by BreastScreen WA that they had dense breasts for the first time (*n* = 1381) or that they had been told (by BreastScreen WA) more than once (*n* = 1309) are collectively referred to as “notified” women (Supplementary Figure S1). To ensure responses truly reflect these defined groups, women who did not fall into the three groups (controls, notified first time, notified multiple times) were excluded from the analysis (*n* = 1421). In addition, women were excluded if they were younger than the BreastScreen WA screening age (<40 years) (*n* = 3), reported previously having a breast cancer (*n* = 378) or having the BRCA mutation (*n* = 24). These additional exclusions and final sample are displayed in the flow chart in Figure 1. Women whose responses were excluded were more likely to be Caucasian/European and have a slightly higher socio-economic status.

Only controls who reported that they have no understanding of what their own breast density to be (*n* = 883; concurrent analysis of this survey question is reported elsewhere [5]) were asked “Knowing my breast density would make me feel… informed/confused/anxious.”

### 2.4. Data Analysis

All data management and quantitative analyses were undertaken using Stata v14. Descriptive statistics were used to describe the characteristics of the three groups (controls, notified for first time, notified multiple times). Proportions were used to describe survey responses by the three groups and compared using Chi-squared tests, where applicable. Women were provided an opportunity to comment on how knowing their breast density made them feel and their future screening intentions. Their responses were analysed thematically through an inductive approach to categorise into a meaningful framework [9]. Responses were coded to the framework and counted (multiple codes per response were permitted).

Ethics and governance approval was obtained from King Edward Memorial Hospital’s Human Research Ethics Committee (#2017046EW RGS000474) and The University of Western Australia (RA/4/20/4178).

## 3. Results

Characteristics of respondents are shown in Table 1. Most respondents were aged between 50 and 74 years, were Caucasian/European, resided in major cities and were generally in higher quintiles of the socio-economic indices. Women who had been notified for the first time were younger than those who had been notified multiple times and the controls.

### 3.1. Self-Reported Psychological Reactions

**Figure 2:**
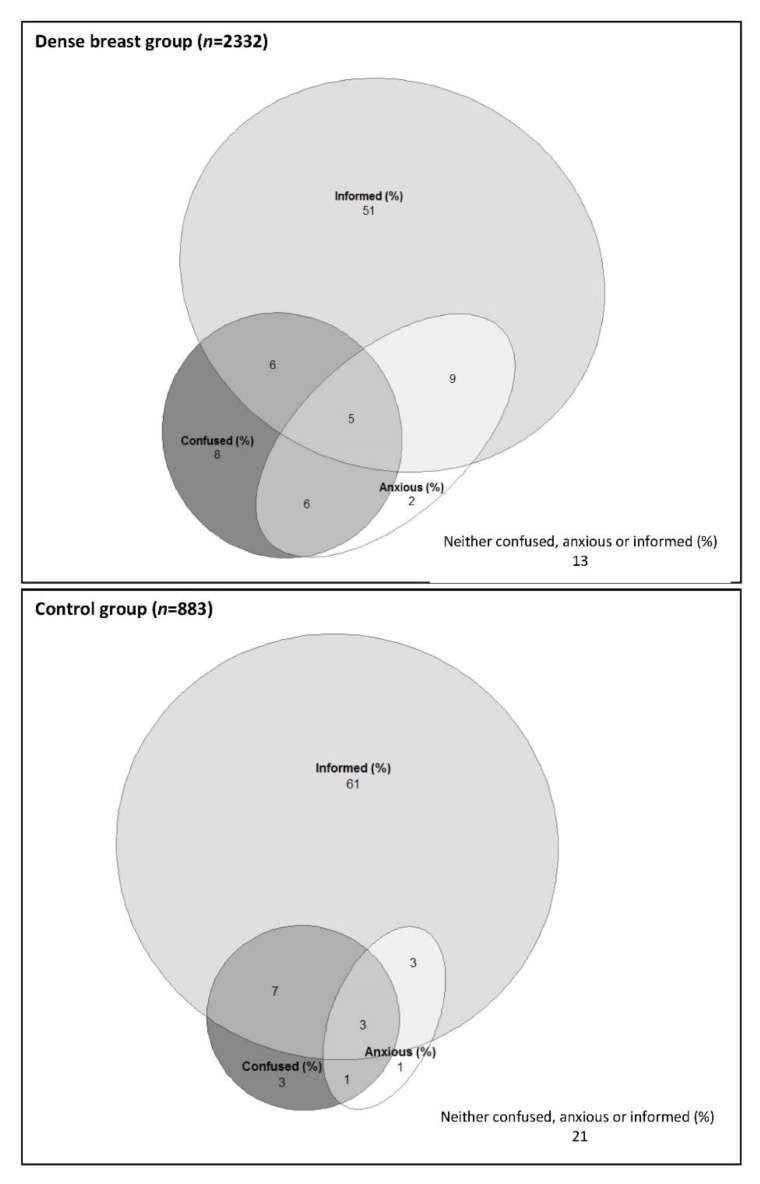
Venn diagrams showing percent of woman reporting feeling informed, confused and anxious for notified women (*n* = 2332) and controls (*n* = 883) (only respondents with complete responses for the three relevant questions are included here).

A summary of the extent women agreed to three statements “Knowing my breast density makes me feel informed/confused/anxious” is displayed in Table 2. Two-thirds of notified women surveyed strongly agreed or agreed that knowing their breast density made them feel informed. Comparatively, over 70% controls indicated that knowing their breast density would make them feel informed (χ^2^ test comparing notified women to control women *p* = 0.005). The proportion of notified women who strongly agreed or agreed with feeling confused was higher in women who were notified for the first time (26%) compared to those who had been notified multiple times (21%) (*p* < 0.001). Similarly, a higher proportion of women notified for the first time strongly agreed or agreed with feeling anxious (23%) than those who had been notified multiple times (18%) (*p* < 0.001). A significantly lower proportion of controls strongly agreed or agreed with feeling confused (13%) or anxious (8%) (*p* < 0.001 comparing controls, women notified for the first time and women notified multiple times). We created an additional respondent grouping which combined responses to feeling anxious and confused into four categories: anxious only (not confused); confused only (not anxious); confused and anxious; and neither confused nor anxious. A majority of notified women were neither anxious nor confused (60%), 11% were anxious only, 13% confused only and 10% both confused and anxious. Controls by comparison reported lower anxiety and confusion, with a higher proportion of controls indicated that knowing their breast density would make them feel neither anxious nor confused (76%), and commensurately lower levels of reporting being anxious only (4%), confused only (9%), or both confused and anxious (4%) (*p* < 0.001). A graphical representation of the overlap between these three groups for both notified women and controls can be seen in Figure 2.

Forty percent of notified women provided comments on their reasons for feeling informed, confused and/or anxious, which informed the coding framework to summarise the types of responses. Comments were categorised into one of five themes: feelings; needs; problem; actions; and not being worried (See Supplementary Table S1 for complete coding framework and example comments). The qualitative framework, collapsed by theme, is presented in Table 3. Of all notified women, 13% described a feeling (6% described feeling anxious, 3% described feeling confused, 4% described being happy to be notified of their breast density, 1% described feeling resigned to the potential increased risk of breast cancer), 8% explained an unmet need, 2% a problem, 7% actions they planned to take and 6% described not being worried. Responses from 7% of women did not fit the coding framework and 59% of women opted not to comment.

Most women noted general comments about their anxiety, for example *“It is unsettling to have a clear mammogram but still be told density can be an issue*”, whereas others specified their anxiety was due to a family history of cancer or the reduced sensitivity of mammography for dense breasts. Although some women said that knowledge of their breast density made them more anxious, some also noted that they appreciated the disclosure of breast density information as it gave them more understanding of their risk factors (e.g., “*I’d rather be a bit anxious because of the information about [breast] density than given a false sense of assurance. My sense of being at above average risk of breast cancer is based on several factors, not solely the density issue*”). Feelings of confusion were mainly about the implications of breast density in relation to breast cancer risk exemplified by comments like, “*I wasn’t aware breast density was a risk factor in developing breast cancer—I just thought the heightened risk was associated with detection.”*

Women who commented that they needed something more in response to breast density notification mainly mentioned a need for general information about breast density (“*I would like to know more about what it means and why I have it*”) or what to do (“*I would like more information - facts and figures; actions to take which are followed up so that I don’t put off any action*. *I don’t know how important it is to do something about this, how often and what to look out for*”). Others mentioned they would like additional or supplemental screening, for example commenting that, “*a mammogram and ultrasound would make me feel more at ease*.”

A small group of women commented to explain concerns they had in relation to mammographic screening. The majority of these mentioned said they had received conflicting advice from different health professionals, or that conversations about breast density had been dismissed. For example, comments included, “*The last dr told me I had a lump, then the specialist I was referred to said know [sic]. So I lack trust in examinations*”, and “*Doctor seemed unconcerned when asked for exam. Said I cd do it myself, But I made appointment*.” A number of women also highlighted concerns about their ability to conduct self-examinations, articulating some common misunderstandings about breast density and the ability to ‘feel’ this trait (e.g., “*Having dense breasts makes me feel a little uneasy about doing my own examination—not knowing what I’m feeling for*.”)

Findings from linking responses to the open-ended question with answers to the closed question on how knowing their breast density made them feel are provided in Table 3. Women who were ‘anxious only’ most commonly left comments explaining their reasons for anxiety (22%). Women who were ‘confused only’ tended to say they needed something additional to what had been provided (18%), this included: more information about breast density; more information about what to do given their breast density; or a need for different or additional screening. Responses from women who were both ‘confused and anxious’ spanned both of these categories, commenting most frequently about their anxiety or needs. Comments provided by women who were ‘neither confused nor anxious’ were most commonly explaining actions they had or would take as a result of being notified (9%), not being worried (9%) or that they were happy to know about their breast density (6%).

Responses gathered from women notified about their breast density for the first time or multiple times were very similar. The only major thematic difference was seen in comments from women who were ‘not confused nor anxious’. More of the women who were notified for the first time than multiple times said they had made or would take further action, most commonly by making a further appointment with a general practitioner (GP) or for additional screening (17.4% and 9.1%, respectively). More women who had been notified of their breast density multiple times than for the first time commented about their lack of worry (16.9% and 10.3%, respectively), explaining this as being due to their lack of family history or other risk factors, that they keep up regular screening practises or that they maintain a healthy lifestyle.

### 3.2. Future Screening Intentions

The results on the future screening intentions of all women are presented in Table 4. Ninety-one percent of notified women said their experience of being told they have dense breasts did not put them off breast screening in the future and that they intended to return for future mammographic screening at BreastScreen WA. Comparatively, 93% of controls and 96% of notified women who responded that knowing their breast density made them feel anxious said they intended to have another mammogram at BreastScreen WA when next due. There was strong evidence of association between collapsed responses (certainly not/likely not, unsure, certainly yes/likely yes, missing) when comparing controls vs. women notified first time vs. women notified multiple times (*p* < 0.0001), and similarly for controls vs. all notified women (*p* < 0.0001), anxious only vs. all notified women (*p* = 0.007), and anxious only women vs. controls (*p* < 0.0001). 

Responses from the 4352 women (82%) who chose to comment on their reasons for their future screening intentions were used to develop a coding framework (See Supplementary Table S2 for complete coding framework and example comments). Eight themes emerged: benefits of screening; perceived susceptibility; social norms; self-efficacy; ease of access; prior behaviour; barriers to screening; and future developments (Table 4). Responses from 158 women (3%) did not fit the coding framework and 972 (17%) chose not to comment.

The most frequently occurring theme of the comments was benefits of screening, with 31% of all women stating it as a reason for their future screening intentions. The potential for early detection of cancer and/or early intervention for treatment, for example, “*It’s the best chance to catch breast cancer in the early stages*” was commonly stated. Women also commented on the sense of reassurance gained from mammographic screening, saying that “*Having mammograms every two years gives me peace of mind about breast cancer*” or it’s “*better safe than sorry*”.

Nearly 15% of women said their intention to return for future screening was the result of a perceived susceptibility to breast cancer. This was attributed mainly to a family history, but some women mentioned a personal history of cancer or breast cancer scare.

A further 9% of women said that the convenience or ease of access to BreastScreen WA was their reason for intending to return for mammographic screening. These women appreciated that the service was offered at no cost, in convenient locations, and that reminders to make an appointment are issued regularly (e.g., “*It’s free quick and easy so why not keep going regularly”* and “*They have a good reminder system and are convenient for me to use*”).

Overall, only 5% of women mentioned barriers to mammographic screening. Some women indicated that they would prefer another type of screening or would attend a different provider who offered different or multiple types of screening. Comments included “*If I was offered an ultrasound instead, I would opt for that*” and “*I will probably have the mammogram at the same place that I have the ultrasound, so that the results can be compared quickly & easily*”. Others noted they were somewhat concerned about the masking effect of breast density in mammography, the associated radiation, or that they would be older than the recommended screening age.

## 4. Discussion

This study found that around a fifth of notified women responded that knowing their breast density made them feel anxious and/or confused; however, their intention to screen again was very high. Qualitative analysis revealed that the source of women’s anxiety was largely due to a family history of breast cancer and the reduced sensitivity of mammographic screening. Women who said they were confused wanted more information about the association between breast density and breast cancer risk, and more information about what to do. Many women understand the benefits of screening and breast density notification does not put the vast majority of women off breast screening in the future.

Yeh and colleagues found that after women received a hypothetical dense breast tissue notification, psychological factors (e.g., anxiety, ambiguity aversion) predicted screening intentions more consistently than demographic factors (e.g., age, minority status) [10]. They found that anxiety mediated the relationship between perceived breast cancer risk and all screening intentions (mammography, ultrasound covered by insurance and ultrasound without insurance coverage). Although perceived risk increased after notification, they concluded that anxiety drives women’s intentions for future screening. Anxiety is a complex emotion, which is influenced by knowledge and attitudes and therefore services concerned that breast density notification increases anxiety should consider what other factors might be influencing anxiety. In this study, the source of most women’s anxiety was related to family history of breast cancer, which, coupled with notification of a second strong risk factor like breast density, represents a group of women who could presumably benefit from consultation with their doctor and continued vigilance with screening. A large proportion of anxious women also made comments about self-efficacy, suggesting that anxiety influences them to be more proactive about breast health rather than reducing action. It is however, also possible that anxiety could also lead to overuse of breast cancer screening and supplemental imaging. Our concurrent investigation of the action women take in response to being notified they have dense breasts shows that overall, 20% of notified women have had an ultrasound due to their breast density [6].

The source of most women’s confusion was largely due to the lack of information available about what to do given their breast density. In the absence of evidence-based screening recommendations for women with dense breasts, confusion about what to do is difficult to combat. In the interim, consistent and clear messaging is vital, particularly as part of the initial notification process, and with potentially more detailed information easily accessible to those with additional questions and concerns.

Reports of anxiety and confusion were more common amongst those who were receiving notification for the first time compared to those who had been notified multiple times, suggesting that anxiety may be decreasing once the knowledge of their density becomes familiar to women. We also found that more women notified for the first time said they had made or would take further action, whereas more women who had been notified multiple times commented about their lack of worry. This finding is consistent with the lessening of anxiety with time for women notified as having dense breasts that was noted by Gunn et al. [11].

Themes that emerged from the qualitative analysis of an open-ended question regarding the reason for women’s future screening intentions were well-aligned with those reported in a previous study of factors associated with screening intentions [12]. It is clear that the benefits of breast cancer screening are widely recognised by women, not just by those who perceive themselves at higher risk but also those who see screening as the social norm. Of the small proportion of women who commented on barriers to future screening, the majority intended to continue screening but said they would prefer an alternative technology or provider.

Limitations of this study include the selection bias introduced by exclusion of those women who did not have an email address. This may result in an under-representation of those from remote or low socio-economic areas. The response rate of 23.5% indicates a large non-response bias; however, the recruitment target was 6000 completed surveys within a 6-month period with a minimum expected participation rate of 10%. We argue that our response rate was good for an unsolicited email invitation for an online survey. Studies have also demonstrated that response rates are not directly correlated with the validity of results, with some studies having response rates as low as 20% yield just as accurate results as studies with response rates of 60–70% [13,14].

In summary, self-reported anxiety following breast density notification within a population-based screening program was relatively low and also appears to increase women’s intentions for future screening, not the reverse. Thus, arguments against breast density notification based on concerns of causing unnecessary anxiety appear to be largely unwarranted, although studies using measures of clinical anxiety are required to confirm this finding.

## Figures and Tables

**Table 1:** Characteristics of respondents who were told they had dense breasts for first time (*n* = 1313), multiple times (*n* = 1194) and who have not been told (*n* = 2817).

Characteristics	Controls (*n* = 2817)	Notified First Time (*n* = 1313)	Notified Multiple Times (*n* = 1194)
Mean age at survey (sd)	61.4 (8.2)	57.6 (9.1)	59.8 (8.0)
Age at survey (%)			
40–49 years	203 (7.2)	258 (19.7)	116 (9.7)
50–74 years	2496 (88.6)	1010 (76.9)	1046 (87.6)
75+ years	118 (4.2)	45 (3.4)	32 (2.7)
Ethnicity (%)			
Caucasian/European	2472 (87.8)	1138 (86.7)	1059 (88.7)
Asian	66 (2.3)	62 (4.7)	40 (3.4)
Aboriginal or Torres Strait Islander	12 (0.4)	2 (0.2)	4 (0.3)
Other	87 (3.1)	48 (3.7)	35 (2.9)
Missing	180 (6.4)	63 (4.8)	56 (4.7)
ARIA ^a^ (%)			
Major city	2257 (80.1)	1094 (83.3)	967 (81.0)
Inner regional	230 (8.2)	83 (6.3)	88 (7.4)
Outer regional	131 (4.7)	46 (3.5)	53 (4.4)
Remote	140 (5.0)	63 (4.8)	68 (5.7)
Very remote	30 (1.1)	14 (1.1)	11 (0.9)
Missing	29 (1.0)	13 (1.0)	7 (0.6)
Education and occupation index ^b^ (%)			
1 (lowest)	334 (11.9)	130 (9.9)	128 (10.7)
2	523 (18.6)	209 (15.9)	220 (18.4)
3	441 (15.7)	184 (14.0)	159 (13.3)
4	572 (20.3)	271 (20.6)	233 (19.5)
5 (highest)	918 (33.6)	506 (38.5)	447 (37.4)
Missing	29 (1.0)	13 (1.0)	7 (0.6)

^a^ Accessibility/Remoteness Index of Australia (ARIA) scores; ^b^ Index of Education and Occupation is on a scale from 1 to 5, where 1 indicates the lowest 20% of the population in Western Australia (least education/occupation opportunities) and 5 indicates the highest 20% of the population in Western Australia (most education/occupation opportunities); Abbreviations: sd, standard deviation.

**Table 2:** Descriptive summary of women’s self-reported psychological reaction to the question “Knowing my breast density makes me feel/would make me feel…” ^a^ for controls (*n* = 883), all notified women (*n* = 2507) and separately for women notified for the first time (*n* = 1313) and for those notified multiple times (*n* = 1194).

	Controls(*n* = 883) ^b^	Notified First Time (*n* = 1313)	Notified Multiple Times (*n* = 1194)	Total Notified (*n* = 2507)
Knowing my breast density makes me feel/would make me feel: ^a^ Informed (%) ^c^				
Strongly disagree	20 (2.3)	31 (2.4)	26 (2.2)	57 (2.3)
Disagree	12 (1.4)	55 (4.2)	48 (4.0)	103 (4.1)
Neither agree or disagree	184 (20.8)	283 (21.6)	254 (21.3)	537 (21.4)
Agree	378 (42.8)	648 (49.4)	617 (51.7)	1265 (50.5)
Strongly agree	250 (28.3)	224 (17.1)	182 (15.2)	406 (16.2)
Missing	39 (4.4)	72 (5.5)	67 (5.6)	139 (5.5)
Confused (%) ^d^				
Strongly disagree	152 (17.2)	176 (13.4)	187 (15.7)	363 (14.5)
Disagree	319 (36.1)	512 (39.0)	497 (41.6)	1,009 (40.3)
Neither agree or disagree	233 (26.4)	208 (15.8)	181 (15.2)	389 (15.5)
Agree	91 (10.3)	287 (21.9)	201 (16.8)	488 (19.5)
Strongly agree	26 (2.9)	53 (4.0)	44 (3.7)	97 (3.9)
Missing	62 (7.0)	77 (5.9)	84 (7.0)	161 (6.4)
Anxious (%) ^d^				
Strongly disagree	207 (23.4)	184 (14.0)	222 (18.6)	406 (16.2)
Disagree	308 (34.9)	499 (38.0)	485 (40.6)	984 (39.3)
Neither agree or disagree	234 (2.7)	242 (18.4)	186 (15.6)	428 (17.1)
Agree	56 (6.3)	273 (20.8)	189 (15.8)	462 (18.4)
Strongly agree	15 (1.7)	34 (2.6)	28 (2.4)	62 (2.5)
Missing	63 (7.1)	81 (6.2)	84 (7.0)	165 (6.6)
Anxious and/or Confused (%) ^d^				
Neither anxious or confused	669 (75.8)	744 (56.7)	758 (63.5)	1502 (59.9)
Anxious only	37 (4.2)	155 (11.8)	113 (9.5)	268 (10.7)
Confused only	83 (9.4)	188 (14.3)	141 (11.8)	329 (13.1)
Anxious and confused	34 (3.9)	152 (11.6)	104 (8.7)	256 (10.2)
Missing	60 (6.8)	74 (5.6)	78 (6.5)	152 (6.1)

^a^ The wording “would make me feel…” was used for controls only; ^b^ Only controls who indicated that they had no idea what their breast density was were asked this question; ^c^ *p*-value from χ^2^ test comparing collapsed responses (strongly disagree/disagree, neither, agree/strongly agree, missing) for controls, notified first time and notified multiple times was 0.05; ^d^ *p*-value from χ^2^ test comparing collapsed responses (strongly disagree/disagree, neither, agree/strongly agree) for controls, notified first time and notified multiple times was <0.001.

**Table 3:** Thematic reasons for psychological reaction for all notified women (*n* = 2507) by four groups defined by quantitative likert responses: Anxious only (*n* = 268), Confused and anxious (*n* = 256), Confused only (*n* = 329), and Not confused or anxious (*n* = 1502). Darker shaded boxes represents most common responses.

Theme	Total (*n* = 2507)	Anxious Only (*n* = 268)	Confused and Anxious (*n* = 256)	Confused Only (*n* = 329)	Not Confused or Anxious (*n* = 1502)	Missing (*n* = 152)
Did not comment (%)	1480 (59.0)	142 (53.0)	114 (44.5)	171 (52.0)	915 (60.9)	138 (90.8)
Commented ^1^ (%)	1027 (41.0)	126 (47.0)	141 (55.1)	158 (48.0)	587 (39.1)	14 (9.2)
Explained feeling anxious	140 (5.6)	59 (22.0)	37 (14.5)	23 (7.0)	21 (1.4)	0 (0.0)
Explained feeling confused	83 (3.3)	3 (1.1)	24 (9.4)	26 (7.9)	30 (2.0)	0 (0.0)
Explained feeling happy to know	89 (3.6)	6 (2.2)	0 (0.0)	1 (0.3)	82 (5.5)	0 (0.0)
Explained feeling resigned	19 (0.8)	0 (0.0)	1 (0.4)	2 (0.6)	16 (1.1)	0 (0.0)
Explained a need	201 (8.0)	29 (10.8)	51 (19.9)	59 (17.9)	61 (4.1)	1 (0.7)
Explained a problem	40 (1.6)	8 (3.0)	10 (3.9)	9 (2.7)	13 (0.9)	0 (0.0)
Explained actions	174 (6.9)	17 (6.3)	10 (3.9)	12 (3.6)	132 (8.8)	3 (2.0)
Explained not being worried	147 (5.9)	4 (1.5)	1 (0.4)	12 (3.6)	130 (8.7)	0 (0.0)
General comment	183 (7.3)	9 (3.4)	17 (6.6)	25 (7.6)	122 (8.1)	10 (6.6)

^1^ Comments could be coded into multiple categories so number in each comment code exceeds overall number of comments.

**Table 4:** Descriptive summary of women’s future screening intentions by breast density notification status (Controls (*n* = 2817), Notified for first time (*n* = 1313) and Notified multiple times (*n* = 1194)) and self-reported psychological reaction to notification for all notified women (Anxious only (*n* = 268), Confused and anxious (*n* = 256), Confused only (*n* = 329), and Not confused or anxious (*n* = 1502)) ^1.^

	Control (*n* = 2817)	Notified First Time (*n* = 1313)	Notified Multiple Times (*n* = 1194)	All Notified (*n* = 2507)				
				Anxious Only (*n* = 268)	Confused and Anxious (*n* = 256)	Confused only (*n* = 329)	Not Confused or Anxious (*n* = 1502)	Missing (*n* = 152)
**Would you say that your experience of being told your breast tissue is dense has put you off breast screening in the future?**								
Definitely not	n/a	1032 (78.6)	991 (83.0)	226 (84.3)	188 (73.4)	248 (75.4)	1329 (88.5)	32 (21.1)
Probably not	n/a	157 (12.0)	96 (8.0)	30 (11.2)	39 (15.2)	54 (16.4)	126 (8.4)	4 (2.6)
Not sure	n/a	27 (2.1)	25 (2.1)	5 (1.9)	15 (5.9)	12 (3.7)	20 (1.3)	0 (0.0)
Yes, probably	n/a	31 (2.4)	23 (1.9)	5 (1.9)	13 (5.1)	12 (3.7)	23 (1.5)	1 (0.7)
Yes, definitely	n/a	2 (0.2)	4 (0.3)	2 (0.8)	0 (0.0)	2 (0.6)	2 (0.1)	0 (0.0)
Missing	n/a	64 (4.9)	55 (4.6)	0 (0.0)	1 (0.4)	1 (0.3)	2 (0.1)	115 (75.7)
**Do you intend to have another mammogram with BreastScreen WA when you are next due? ^2^**								
Certainly not	2(0.1)	2 (0.2)	4 (0.3)	0 (0.0)	1 (0.4)	1 (0.3)	4 (0.3)	0 (0.0)
Likely not	15 (0.5)	18 (1.4)	5 (0.4)	2 (0.8)	7 (2.7)	5 (1.5)	9 (0.6)	0 (0.0)
Unsure	11 (0.4)	44 (3.4)	24 (2.0)	9 (3.4)	12 (4.7)	17 (5.2)	29 (1.9)	1 (0.7)
Likely yes	188 (7.0)	181 (13.8)	112 (9.4)	40 (14.9)	41 (16.0)	55 (16.7)	154 (10.3)	3 (2.0)
Certainly yes	2425 (86.41)	1003 (76.4)	994 (83.3)	216 (80.6)	194 (75.8)	249 (75.7)	1305 (86.9)	33 (21.7)
Missing	176 (6.3)	65 (5.0)	55 (4.6)	1 (0.4)	1 (0.4)	2 (0.6)	1 (0.1)	115 (75.7)
**Can you comment on the reason for your intention?**								
Did not comment	361 (12.8)	356 (27.1)	255 (21.4)	50 (18.7)	61 (23.8)	75 (22.8)	301 (20.0)	124 (81.6)
Commented ^1^	2456 (87.2)	957 (72.9)	939 (78.6)	218 (81.3)	195 (76.2)	254 (77.2)	1201 (80.0)	28 (18.4)
Benefits of screening	1052 (32.1)	320 (24.4)	305 (25.5)	85 (31.7)	61 (23.8)	84 (25.5)	384 (25.6)	11 (7.2)
Perceived susceptibility	534 (19.0)	79 (6.0)	143 (12.0)	32 (11.9)	33 (12.9)	25 (7.6)	129 (8.6)	3 (2.0)
Social norm/influence	329 (11.7)	209 (15.9)	178 (14.9)	33 (12.3)	29 (11.3)	49 (14.9)	269 (17.9)	7 (4.6)
Self-efficacy	227 (8.1)	149 (11.3)	129 (10.8)	37 (13.8)	20 (7.8)	25 (7.6)	192 (12.8)	4 (2.6)
Ease of access	267 (9.5)	84 (6.4)	115 (9.6)	17 (6.3)	11 (4.3)	28 (8.5)	140 (9.3)	3 (2.0)
Prior behaviour	199 (7.1)	51 (3.9)	47 (3.9)	5 (1.9)	7 (2.7)	19 (5.8)	65 (4.3)	2 (1.3)
Barriers to screening	54 (1.9)	111 (8.5)	80 (6.7)	25 (9.3)	40 (15.6)	40 (12.2)	85 (5.7)	1 (0.7)
Future developments	2 (0.1)	21 (1.6)	10 (0.8)	4 (1.5)	1 (0.4)	6 (1.8)	20 (1.3)	0 (0.0)
General comment	87 (3.1)	35 (2.7)	36 (3.0)	6 (2.2)	10 (3.9)	15 (4.6)	40 (2.7)	0 (0.0)

^1^ Comments could be coded into multiple categories so number in each comment code exceeds overall number of comments. ^2^ *p*-values from χ^2^ tests comparing collapsed responses (certainly not/likely not, unsure, certainly yes/likely yes, missing) for controls, notified first time and notified multiple times *p* < 0.0001, for controls vs. all notified women *p* < 0.0001, for anxious only vs. all notified *p* = 0.007, for anxious only vs. controls *p* < 0.0001.

## Supplementary Materials

The following are available online at https://www.mdpi.com/2077-0383/9/4/955/s1.
