# Supplementary material for: Confusion and Anxiety Following Breast Density Notification: Fact or Fiction?
Source: J Clin Med. 2020 Mar 30;9(4):955. doi: 10.3390/jcm9040955 (PMC7230559; doi:10.3390/jcm9040955)
Supplement: Supplementary file 1 [file jcm-09-00955-s001.pdf]

Supplementary Figure 1. Sample results letter. Online only.

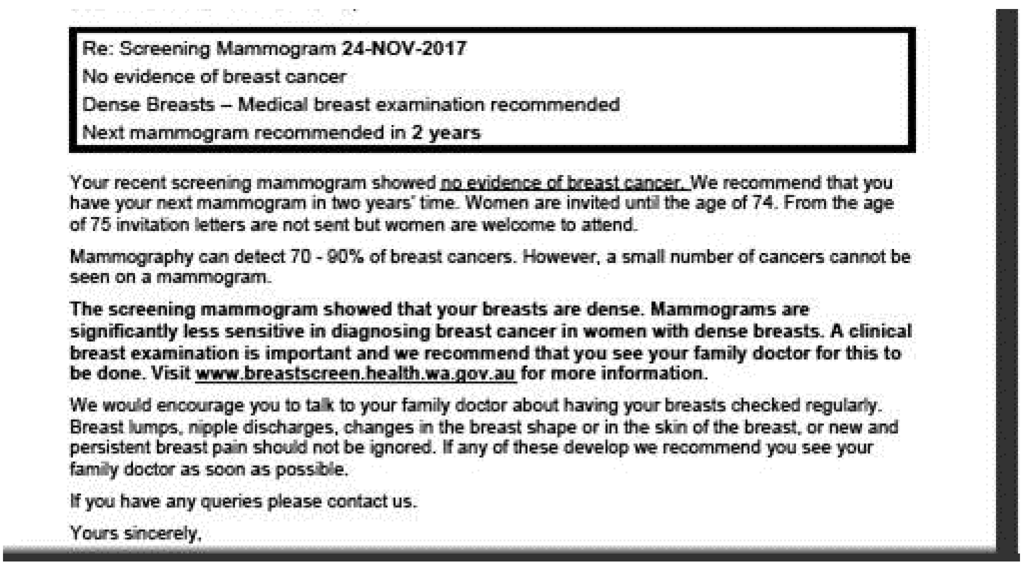

Re: Screening Mammogram **24-NOV-2017**
No evidence of breast cancer
Dense Breasts – Medical breast examination recommended
Next mammogram recommended in **2** years

Your recent screening mammogram showed no evidence of breast cancer. We recommend that you have your next mammogram in two years' time. Women are invited until the age of 74. From the age of 75 invitation letters are not sent but women are welcome to attend.

Mammography can detect 70 - 90% of breast cancers. However, a small number of cancers cannot be seen on a mammogram.

The screening mammogram showed that your breasts are dense. Mammograms are significantly less sensitive in diagnosing breast cancer in women with dense breasts. A clinical breast examination is important and we recommend that you see your family doctor for this to be done. Visit www.breastscreen.health.wa.gov.au for more information.

We would encourage you to talk to your family doctor about having your breasts checked regularly. Breast lumps, nipple discharges, changes in the breast shape or in the skin of the breast, or new and persistent breast pain should not be ignored. If any of these develop we recommend you see your family doctor as soon as possible.

If you have any queries please contact us.

Yours sincerely,

Supplementary Figure 2: Flow chart of breast density survey respondents

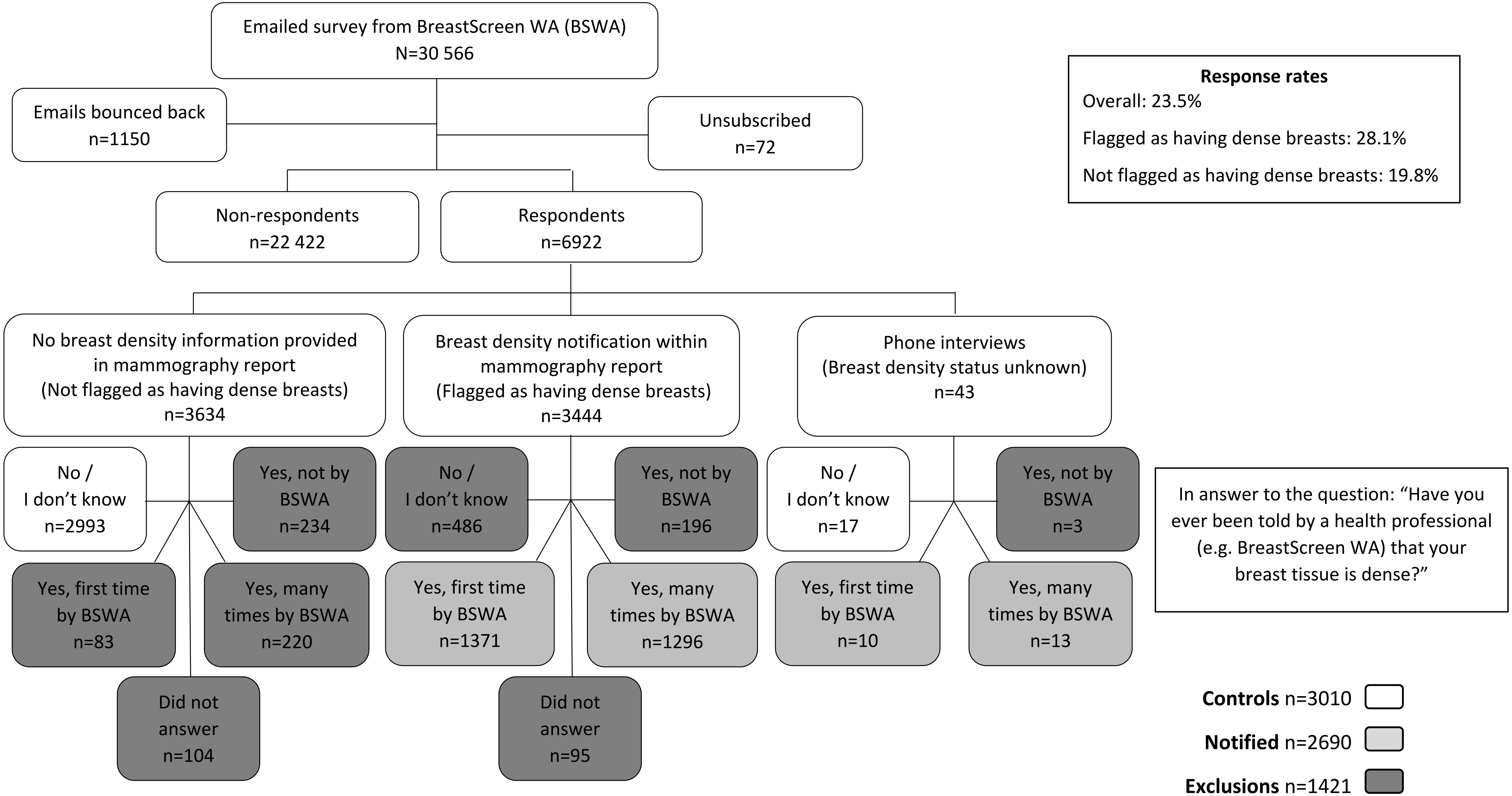

Supplementary Table 1: Framework developed from qualitative analysis of open-ended responses from notified women who commented on the reason(s) for their emotional reaction (feeling informed, confused, and/or anxious) with number and percentage of comments (n=1,027) falling within each code.

Theme	Category	Code	Total comments (n=1,027)	Example quote
Explained feelings	Anxious	General worry	56 (5.5)	*"Knowing that I have dense breasts gives me more knowledge but also makes me a little anxious that I know I have a greater chance of getting breast cancer."*<br>*"I'd rather be a bit anxious because of the information about [breast] density than given a false sense of assurance. My sense of being at above average risk of breast cancer is based on several factors, not solely the density issue."*
		Due to family history	40 (3.9)	*"Two maternal aunts had breast cancer ... maybe my chances are genetically higher...?"*<br>*"Anxiety levels high as have a close family member who has had a masectomey [sic] recently"*
		Due to poor sensitivity of mammography	27 (2.6)	*"My main concern is due to density of tissue there could be undetected breast cancer"*
		While waiting for further appointment (e.g. GP, additional screening)	13 (1.3)	*"I was a bit anxious until I had my clinical breast examination by GP and it was all good."*<br>*"I will have an ultra sound in the next week so I am feeling a bit anxious about that."*
		Due to personal history (breast or other cancer)	4 (0.4)	*"A feel a little anxious because I have had endometrial cancer previously."*
		Total Anxious	140 (13.6)	
	Confused	About breast density and cancer risk	45 (4.4)	*"I wasn't aware breast density was a risk factor in developing breast cancer - I just thought the heightened risk was associated with detection."*<br>*"I would love to know more about what breast density means, its implications and what I should do about it."*
		About why am I being told now?	22 (2.1)	*"Despite mammograms over the last 30 years and several exams by GP, this was the first time I had been told my breasts were dense"*<br>*"I am confused as to why this has never been mentioned before - have all the previous mammograms been a waste of time?"*
		About what is breast density	12 (1.2)	*"I had a breast exam after I was told I had breast density and all fine. I was not given any information on breast density at the time of my mammogram. I know nothing about breast density."*
		About the impact of HRT	4 (0.4)	*"On HRT patches unsure if this can increase breast density."*
		Total Confused	83 (8.1)	
	Happy to know because	Awareness is important	60 (5.8)	*"Knowledge assists with making informed decisions"*<br>*"It concerns me that there is a problem but I feel that the knowledge is a good thing"*<br>*"I feel informed and good that I am aware of this"*
		I can be more proactive	29 (2.8)	*"I am pleased that I have been informed so in future I can be more self aware & follow through with monitoring"*<br>*"Knowing that I have dense breasts has made me take responsibility to have further regular testing for breast cancer"*
		Total Happy to know because	89 (8.7)	
	Resigned	Nothing can be done	19 (1.8)	*"Nothing I can do about it so no point worry[ing] - just hope that if it developed within 2 year periods, it is picked up during a mammogram"*<br>*"I feel I have this information however there is nothing I can do to reduce my risk with density"*
		Total Resigned	19 (1.8)	
	Total Explained feelings		331 (32.2)	

Explained needs	Need something more	General information	78 (7.6)	*"I would like more information about the impact breast density can have"*<br>*"I would like to know more about what it means and why I have it"*
		Additional/different screening	52 (5.1)	*"A mammogram and ultrasound would make me feel more at ease."*<br>*"I feel further tests need to be offered for women with high breast density"*
		Information about the process/what to do	51 (5.0)	*"I don't mind knowing - as long as I know what to do extra to increase the chance of picking up issues."*<br>*"I would like more information - facts and figures; actions to take which are followed up so that I don't put off any action. I don't know how important it is to do something about this, how often and what to look out for."*
		Specialist advice	12 (1.2)	*"I will discuss my results with my GP to ensure I have stronger understanding about this subject & impact on me"*<br>*"I need to be better informed on this issue & will discuss with my GP."*
		Personal advice (how does it affect me)	8 (0.8)	*"I would like to know how dense. I read it's graded in some country's. It would give you more awareness."*<br>*"Would be beneficial to be told how dense my breasts actually are"*
	Total Explained needs		201 (19.6)	
Explained actions	Actions	Have booked/had a further appointment	108 (10.5)	*"About to follow up with GP regarding my last screen as it always mentions density, will have an examination and discuss further…"*<br>*"I am better informed having seen my GP and will attend an ultrasound"*
		Will continue with regular screening	52 (5.1)	*"I have regular breast screening and will now have examinations regularly by my GP"*<br>*"I will keep checking my breasts and go and see the doctor more"*
		Other (eg self check)	14 (1.4)	*"Probably be more aware of doing more self check on breast"*<br>*"After checking dense breast information on the internet, I think I would benefit from weight loss, as well as a check by my GP."*
	Total Explained Actions		174 (16.9)	
Explained not being worried	Not worried because	Generally not worried	50 (4.9)	*"Personal attitude - being positive and realistic about this situation, dense breasts are not all doom and gloom."*<br>*"Knowing my breast density hasn't altered my thinking in any way"*
		I have no family history/risk factor	33 (3.2)	*"Have never worried too much as being [a bit] a complacent I guess as breast cancer does not run in my family."*
		I am healthy / I have a healthy lifestyle	29 (2.8)	*"Aware but not alarmed as I try to stay healthy"*<br>*"I don't feel I'm likely to get breast cancer for some reason, have other serious health issues, but am not a smoker, drinker and don't have any relative who has had breast cancer"*
		I screen regularly	24 (2.3)	*"I realise dense breast may increase risk of breast cancer, but if I have regular self checks, and mammogram checkups with follow up Dr appointments, that's about all I can do for myself"*<br>*"I feel very confident because of the regular visits to the breastscreen mobile unit. Regardless of diagnosis..knowledge is power."*
		I was reassured by my GP	11 (1.1)	*"I feel confident in how the breast screening and Doctor handled my situation with dense breasts"*<br>*"I am not worried because my GP told me not to after he examined my breasts"*
	Total Explained not being worried		147 (14.3)	
Explained a problem	Problem	Received conflicting advice/dismissive response	22 (2.1)	*"My report said dense breasts 2 years ago, but this year the report did not mention that they were dense, so I'm a bit confused if they are less dense or if the radiologist has not interpreted them as dense this time"*<br>*"The last dr told me I had a lump, then the specialist I was referred to said know [sic]. So I lack trust in examinations"*
		Self exams are difficult	12 (1.2)	*"Having dense breasts makes me feel a little uneasy about doing my own examination - not knowing what I'm feeling for."*

	Prefer a different source of advice/information	6 (0.6)	*"I feel that because my breasts are dense it is harder to detect abnormalities both during a self examination and during clinical tests or screens"* *"I am hesitant to research breast density on the internet until I have a reliable source/website."* *"It seems more difficult to find a Dr that I feel comfortable following up with. It would be great if breastscreen had a female Dr attached to the site for examinations. More likely to make an appointment."*
	Total Explained a problem	40 (3.9)	
General comments		294 (28.6)	

Note: some comments were coded multiple times; cumulative count of codes does not equal total comments

Abbreviations: GP; General Practitioner. HRT; Hormone Replacement Therapy

Supplementary Table 2: Coding framework for qualitative analysis of women's intentions for future mammographic screening with BreastScreen Western Australia with number and percentage of comments (n=4,352) falling within each code.

Theme	Code	Total comments (%) (n=4,352)	Example comments
Benefits of screening	Early detection / Intervention	726 (16.7)	*"I believe that if I have breast cancer, the sooner it's caught, the more likely it is that it can be treated successfully."* *"Possibility of early detection if there are issues"*
	Reassurance / Be safe / Make sure no cancer	410 (9.4)	*"Better to be safe than sorry"* *"For peace of mind to know that my breasts are not showing signs of cancer."*
	Prevention of cancer	207 (4.8)	*"Because prevention is better than a cure!"* *"Strongly believe in preventing illness not trying to cure it! Though I feel I am low risk will still have another test in 2yrs"*
	To detect cancer / Monitor health/changes	187 (4.3)	*"I do it in the hope that there is a chance of picking up on an abnormality in the breast."* *"I will continue to have examinations to be sure of any changes."*
	Screening is recommended despite breast density obscuring cancer	126 (2.9)	*"Because my understanding is that, whether dense or not, screening is the best detection."* *"Will continue to monitor this way as well as physical examination until there is better way."*
	Not confident with self-examination	11 (0.3)	*"I don't very often check my breasts for lumps as I'm not sure if I'm doing it properly, so I rely on the mammograms."* *"I have large breasts that I have trouble doing self examination....too much area to cover! I'm not confident on finding cancer by myself."*
	For the greater good / Benevolence	10 (0.2)	*"And all scans help toward better research and treatment for the future population of young females."* *"Supporting a state funded health program and contributing to women's health"*
	Total Benefits of screening	1,677 (38.5)	
Perceived susceptibility	Family history (breast cancer or other cancer)	574 (13.2)	*"I intend to have regular mammograms as my mother died of breast cancer so I intend to be vigilant."* *"With a history of Breast Cancer in my family I would prefer to take every possible precaution."*
	Personal history (breast cancer or other cancer/scare)	85 (2.0)	*"I had breast cancer in 2001 and, having dense breast tissue also, I feel it is common sense to have a mammogram yearly."* *"Breast cancer was picked up by a screen"*
	Age	52 (1.2)	*"Incidence of breast cancer increases with age"* *"I think it is necessary at my age"*
	Other (eg breast density, cysts)	26 (0.6)	*"Because I have never breast fed and also have sensitive breasts."* *"Why would being informed of density dissuade me from a mammogram? If anything it would further encourage me."*
	HRT use	19 (0.4)	*"Because I take hormonal replacement medication I believe I should have the mammogram each year"*
	Total Perceived susceptibility	756 (17.4)	
Social norm / Influence	Screening is important / General intend to screen	243 (5.6)	*"I believe it is important to continue to be monitored through regular mammograms."* *"Due to the nature of my breasts I understand the importance of having regular mammograms."*
	Common sense / Better than nothing / Why not	225 (5.2)	*"Be silly not to"* *"It's the sensible thing to do and may save my life"*
	Screening is recommended / The done thing / I'm told to (eg reminder letter, partner)	178 (4.1)	*"My GP recommends screen every 2 years"* *"Because I get a reminder"*

	Friend/colleague affected by breast cancer	70 (1.6)	*"Observation of the number of people I know who have had breast cancer even over 80 years of age."*<br>*"I have had at least 5 friends die from breast cancer, I have friends who are survivors and I have a second cousin who has it."*
	Total Social norm / Influence	716 (16.5)	
Self-efficacy	My health is important	203 (4.7)	*"I think it is good to take any precautions available to monitor my breast health"*<br>*"Breast screening is another tool available for use in looking after my breast health so I will continue with mammograms."*
	I want to be informed/aware	141 (3.2)	*"By having regular mammograms allows me to make an informed decision about my breasts health"*<br>*"Better to be aware than do the ostrich thing and put your head in the sand. Or... what you don't know can't hurt you."*
	Proactive healthcare	99 (2.3)	*"So that I can take action if something unusual shows up."*<br>*"It is best to be proactive when it comes to breast cancer screening."*
	Precaution / Risk management	62 (1.4)	*"I think it is good to take any precautions available to monitor my breast health"*<br>*"I believe in taking as many precautions as I can with my health and the free breast screening is one."*
	Total Self-efficacy	505 (11.6)	
Ease of access	Convenience / Cost / Service / Reminder	466 (10.7)	*"Regular and free screening at a convenient location is a no-brainer and is a responsible thing to do for maintaining health."*<br>*"Free service, reasonable chance of detecting a cancer if present"*
	Total Ease of access	466 (10.7)	
Prior behaviour	Routine/regular check-ups	297 (6.8)	*"I have a mamogram every 2 years so will continue"*<br>*"though trust I am unlikely to have breast cancer, happy to continue the mammogram every two years."*
	Total Prior behaviour	297 (6.8)	
Barriers to screening	Prefer another provider/type of screening	83 (1.9)	*"I think I need an ultrasound/MRI if regular Mammograms aren't showing much, and a private clinic can offer this in one appt."*<br>*"I rather be vigilant, however I would request an ultrasound as well as I've been told mammograms are not the best way to detect cancer in dense breasts."*
	Mammography is not effective / Detection is not sufficient	41 (0.9)	*"I'm now wondering if mammogram is a waste of time, since my dense breast tissue looks like cancer. I worry that my lumpy breasts could be hiding cancerous lumps. I would like an ultrasound or PET scan but figure that won't happen unless I have a discrete lump. So, I worry that something might be missed."*<br>*"Well if they couldn't see because of the density issue it would be the same problem if I got it done again"*
	Over the age for screening	29 (0.7)	*"It will probably be my last breast density test as I will be about75 yrs old."*<br>*"I shall be too old to get a reminder, so I'll have to remember to make the appointment!"*
	Need specialist advice	20 (0.5)	*"I still think it's a good service and a 2 yearly check is good. I will however seek other advice regarding ultra sound."*<br>*"Need to get further advice from the dr."*
	Concern about radiation	15 (0.3)	*"If I find and abnormality, then yes I will need to attend prior to my yearly scan. I do worry about the radiation each year though."*<br>*"If mammogram is not able to pick up cancer in dense breasts... wonder if the radiation exposure risk is sensible"*
	Had a bad experience	15 (0.3)	*"I find having a mammogram immensely painful - I believe this is related to my breast density. The breast screeners seem unaware of how painful this is, so I would prefer a method which is not so painful and will investigate this in the future."*<br>*"Hate them, but they can pick up early changes"*
	Want more information	15 (0.3)	*"I do not know what it means to have dense tissue breasts. I shall now have to make time to find out."*<br>*"I'm actually unsure as to the affect dense breasts has with regard to breast cancer so I really need more information about this. I'm definitely going to continue attending breast screen every 2 years."*

	Other barriers	11 (0.3)	*"I will continue to have a mammogram, but unclear as to whether there is any advantage in doing so"*<br>*"I will probably leave it longer than the two years. I had three years between my last ones"*
	Distance / Relocating	11 (0.3)	*"I would look to have the mammogram if we are in town at the time the mobile clinic is here."*
	No risk factors	5 (0.1)	*"I have a healthy diet (high plant foods with some animal proteins and fats, some alcohol, no smoking) and lifestyle (moderate exercise almost daily) with no family history of breast cancer"*<br>*"My mother (include other female family) has not got it."*
	Total Barriers to screening	245 (5.6)	
Future developments	My breasts/density may change	25 (0.6)	*"I'm going to my next appointment because my breasts density may have reduced over the 12mths."*<br>*"I was told that breasts generally become less dense due to age."*
	Improvements in technology / Knowledge of breast density	8 (0.2)	*"Maybe doctors will know more about dense breast tissue and how to read whether there is cancer in it with the mammograms by the time my Breast screen comes about."*<br>*"Medical science & equipment improves all the time."*
	Total Future developments	33 (0.8)	
General comments		158 (3.6)	

Note: some comments were coded multiple times; cumulative count of codes does not equal total comments
